# From Osteoclast Differentiation to Osteonecrosis of the Jaw: Molecular and Clinical Insights

**DOI:** 10.3390/ijms20194925

**Published:** 2019-10-04

**Authors:** Alexandre Anesi, Luigi Generali, Laura Sandoni, Samantha Pozzi, Alexis Grande

**Affiliations:** 1 Department of Medical and Surgical Sciences for Children & Adults, University of Modena and Reggio Emilia, Via del Pozzo 71, 41124 Modena, Italy; alexandre.anesi@unimore.it; 2 Department of Surgery, Medicine, Dentistry and Morphological Sciences with Transplant Surgery, Oncology and Regenerative Medicine Relevance, University of Modena and Reggio Emilia, 41121 Modena, Italy; luigi.generali@unimore.it; 3 Department of Biomedical, Metabolic and Neural Sciences, University of Modena and Reggio Emilia, Via Giuseppe Campi 287, 41125 Modena, Italy; 196042@studenti.unimore.it

**Keywords:** osteoclast differentiation, anti-resorptive therapy, bisphosphonates, denosumab, osteonecrosis of the jaw

## Abstract

Bone physiology relies on the delicate balance between resorption and formation of its tissue. Bone resorption depends on a process called osteoclastogenesis in which bone-resorbing cells, i.e., osteoclasts, are produced by the differentiation of more undifferentiated progenitors and precursors. This process is governed by two main factors, monocyte-colony stimulating factor (M-CSF) and receptor activator of NFκB ligand (RANKL). While the former exerts a proliferating effect on progenitors/precursors, the latter triggers a differentiation effect on more mature cells of the same lineage. Bone homeostasis requires a perfect space–time coordination of the involved signals. When osteoclastogenesis is poorly balanced with the differentiation of the bone forming counterparts, i.e., osteoblasts, physiological bone remodelling can turn into a pathological state, causing the systematic disruption of bone tissue which results in osteopenia or osteolysis. Examples of these conditions are represented by osteoporosis, Paget’s disease, bone metastasis, and multiple myeloma. Therefore, drugs targeting osteoclastogenesis, such as bisphosphonates and an anti-RANKL monoclonal antibody, have been developed and are currently used in the treatment of such diseases. Despite their demonstrated therapeutic efficacy, these agents are unfortunately not devoid of side effects. In this regard, a condition called osteonecrosis of the jaw (ONJ) has been recently correlated with anti-resorptive therapy. In this review we will address the involvement of osteoclasts and osteoclast-related factors in the pathogenesis of ONJ. It is to be hoped that a better understanding of the biological mechanisms underlying bone remodelling will help in the design a medical therapeutic approach for ONJ as an alternative to surgical procedures.

## 1. Osteoclast Differentiation

### 1.1. A General Overview of Osteoclast Differentiation

**Figure 1:**
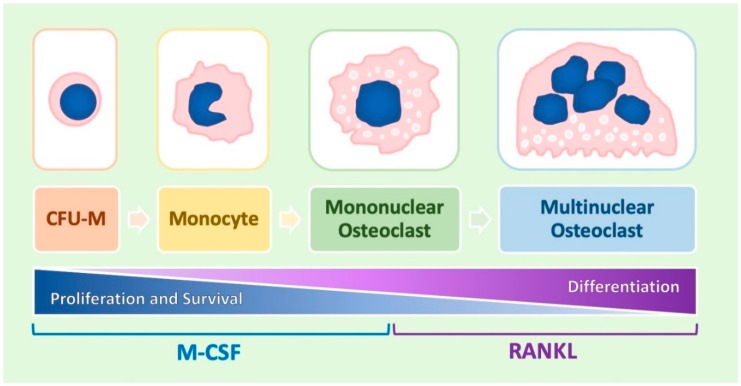
The different steps of osteoclast differentiation. Osteoclastogenesis consists of four main steps, represented in the figure from the left to the right: colony forming unit-monocyte (CFU-M), monocyte, mono-nuclear osteoclast, and multinuclear osteoclast. The initial phases of the process are supported by monocyte-colony stimulating factor (M-CSF), mainly exerting a proliferation/survival effect, whereas the remaining events are stimulated by ligand of receptor activating NFκB (RANKL), promoting a terminal differentiation effect.

Osteoclasts (OCs) are bone-resorbing cells that belong to the monocyte/macrophage lineage and are produced through a differentiation process called osteoclastogenesis (Figure 1). The first cell appearing during osteoclastogenesis is represented by the colony forming unit-monocyte (CFU-M), a progenitor that resides in bone marrow and originates from the pluripotent hematopoietic stem cell [1,2]. The CFU-M gives rise to more differentiated precursors called monoblasts, which in turn differentiate to monocytes. This initial phase of osteoclastogenesis is also called monocyte differentiation or monocytopoiesis. After their release in the blood stream, monocytes migrate to the bone tissue where they are transformed into mononuclear OCs (in other terms, bone tissue macrophages). These cells can then fuse together, forming multi-nucleated OCs [1,2]. The functional difference between mononuclear and multi-nucleated OCs is not exactly clear but it is conceivable that the latter are characterized by a more pronounced capacity to induce bone resorption as compared to the former.

### 1.2. Molecular Regulation of the Process

The molecular regulation of osteoclast differentiation is quite complex (Table 1) and, starting from this premise, attention will be especially focused on aspects exhibiting evident physio-pathological and therapeutic implications that are strictly related to the aims of the review.

The correct fulfilment of osteoclastogenesis requires the presence of a couple of important signalling molecules. The first is represented by monocyte colony-stimulating factor (M-CSF), exerting a proliferation effect on the early pre-monocyte phase of the process and a survival effect on the entire process [3,4]. The second is represented by the ligand of receptor activating NFκB (RANKL), responsible for the differentiation effect occurring in the late post-monocyte phase of the process that is necessary to transform monocytes into OCs [3,4].

### 1.3. Role Played by M-CSF

**Figure 2:**
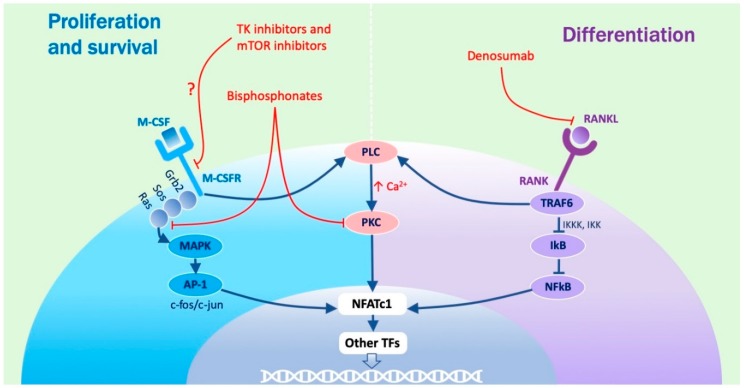
The molecular regulation of osteoclast differentiation. Osteoclast formation is regulated by M-CSF and RANKL. M-CSF binds to its receptor (M-CSFR), a tyrosine kinase receptor encoded by the *c-fms* gene, triggering the mitogen activated protein kinase (MAPK) phosphorylation cascade and leading to the activation of the activator protein -1 (AP-1) transcription factor which up-regulates the expression of genes promoting cell proliferation. On the other hand, RANKL, acting through its receptor RANK, inhibits inhibitor of NFκB (IκB) which sequesters nuclear factor kappa-light-chain-enhancer of activated B cells (NFκB) under basal conditions, thus leading to the consequent release and nuclear translocation of this transcription factor. Once activated, NFκB induces the expression of nuclear factor of activated T-cells cytoplasmic 1 (NFATc1)—the master regulator of osteoclastogenesis), the activity of which is also aided by AP-1. Both M-CSFR and RANK are able to stimulate the phospholipase C (PLC) – protein kinase C (PKC) pathway, which contributes to NFATc1 activation. The final and common effect of the three pathways is represented by an increased transcription of osteoclast differentiation markers. Anti-resorptive agents and their molecular targets are indicated in red inside the figure. TK, tyrosine kinase; mTOR, mammalian target of rapamycin. Arrows mean activation, T bars mean inhibition.

The biological effects of M-CSF are determined through a typical tyrosine kinase receptor pathway (Figure 2).

In fact, M-CSF induces the dimerization, phosphorylation, and activation of its receptor (M-CSFR), encoded by the *c-fms* gene that, acting through the Grb2 and Sos adapter proteins and the Ras G protein, triggers the activation of a family of serine/threonine kinases that are called mitogen activated protein kinase (MAPK). MAPK are then responsible for the phosphorylation and activation of the activator protein—1 (AP-1) transcription factor, resulting by the dimerization of the proteins encoded by the *c-jun* and *c-fos* genes and, once formed, the AP-1) complex moves to the nucleus where it activates the transcription of target genes promoting cell division. The proliferation effect of M-CSF is also at least partly mediated by a cytosolic non-receptor tyrosine kinase that is called c-src (not represented in Figure 2).

On the other hand, the phosphorylated tyrosine kinase receptor also activates phosphatidyl inositol 3 kinase (PI3K) which in turn leads to the activation of two serine/threonine kinases that are named AKT and mammalian target of rapamycin (mTOR). These enzymes are then respectively responsible for the survival and the metabolic effects of M-CSF (not represented in Figure 2).

### 1.4. Role Played by RANKL

RANKL determines its effects acting through the so-called nuclear factor kappa-light-chain-enhancer of activated B cells (NFκB) pathway. In this pathway, RANKL binds to its cognate receptor, RANK, inducing the intra-cellular recruitment of a family of proteins called TNF receptor associated factors (TRAF), among which the most important member is represented by TRAF6. Acting through a cascade of serine/threonine kinases named IκB kinase kinase (IKKK) and IκB kinase (IKK), TRAF6 determines the phosphorylation and degradation of a protein named inhibitor of NFκB (IκB) which, under basal conditions, sequesters and inactivates the NFκB transcription factor. NFκB becomes consequently stabilized and migrates to the nucleus where it activates the transcription of its target genes [5]. The most important among them is represented by the gene coding for the nuclear factor of activated T-cells cytoplasmic 1 (NFATc1) transcription factor [6,7,8], which is the master regulator of osteoclast differentiation and is responsible for the up-regulated expression of virtually all osteoclast differentiation markers, including dendritic cell-specific transmembrane protein (DC-STAMP), H+ ATPase, tartrate-resistant acid phosphatase (TRAP), cathepsin K (CSTK), and matrix metallo-protease 9 (MMP9) [9].

### 1.5. Role of Osteoclast Differentiation Markers

Once expressed, osteoclast differentiation markers are responsible for the acquisition of all the typical phenotypic and functional properties of OCs. More precisely, DC-STAMP mediates cell fusion, and therefore the formation of multi-nucleated OCs; H^+^ ATPase and TRAP are responsible for the degradation of the mineral (inorganic) component of bone; and CSTK and MMP-9 are responsible for the degradation of the protein (organic) component of bone.

### 1.6. Transcription Factors Regulating NFATc1 Activity

NFATc1 activity is due to a large set of transcription factors that are recruited by NFATc1 itself and favour the activation of its target genes. Examples of such transcription factors are represented by c-fos (already mentioned for its participation to the AP-1 complex [10]), PU.1 (also involved in the regulation of early monocyte differentiation [11]), various members of the microphthalmia-associated transcription factor (MitF) family (such as TFE3 [12,13,14]), and MafB, although with apparently contradictory observations [15,16,17,18,19].

The B-cell lymphoma 6 (Bcl6) transcription factor, conversely, antagonizes NAFTc1 activity and suppresses osteoclastogenesis [20].

### 1.7. Role Played by PKC

The molecular effects of NFATc1 and the other cooperating transcription factors is strongly supported by the phosphorylation activity of a Ca^2+^-dependent serine/threonine kinase called protein kinase C (PKC) [21,22]. This kinase is activated following the intra-cellular increase of Ca^2+^ concentrations that is normally promoted by the activation of phospholipase C (PLC). In the situation under consideration, both the involved signalling pathways can activate PLC. In fact, in the case of M-CSF, PLC activation is determined by a direct interaction with the phosphorylated tyrosine receptor, whereas in the case of RANKL, the same effect is reached through a recruitment operated by TRAF6. These molecular mechanisms explain why agonists of the PLC–PKC pathway are able to sensibly potentiate osteoclast differentiation, whereas its inhibitors do exactly the opposite.

### 1.8. Role Played by Vitamin D3

Although controversial, a remarkable number of observations, listed below, support the role played by the active form of vitamin D3 (VD3) in the activation of osteoclast differentiation [23,24]. In fact: (1) stimulation with VD3 induces the monocyte differentiation of a large variety of hematopoietic cell types, suggesting that, although indirectly, it favours osteoclast differentiation [25,26,27,28,29]; (2) treatment of the U937 monocytic cell line with VD3 and a powerful agonist of PKC, named phorbol myristate acetate (PMA), induces osteoclast differentiation [30]; (3) exposure of osteoblasts to VD3 activates the production of RANKL, the main inducer of osteoclast differentiation [31,32]; (4) acting on several cell types including OCs, VD3 induces the secretion of osteopontin, promoting the adhesion of OCs to extra-cellular matrix inside bone tissue [33,34]; (5) treatment of RANKL activated monocytes with VD3 potentiates osteoclast differentiation, although this effect favours the formation of mono-nucleated elements in place of their multi-nucleated counterpart [35,36]; and (6) knock-out of the vitamin D3 receptor (VDR), mediating the effect of VD3, abrogates osteoclast differentiation [37]. The apparent contrast between the capacity to activate OCs described thus far and the overall effect of bone deposition promoted by VD3 on bone tissue might be explained by the consideration that VD3 probably acts by stimulating the so-called bone remodelling cycle, responsible for the replacement of old bone with new one (see below) [38].

### 1.9. Role of Cytokines, Macrophage Polarization, and Chemical Factors

In addition to M-CSF and RANKL, numerous other signalling molecules, all classified in the cytokine family, regulate osteoclastic differentiation by either favouring or inhibiting the process [39]. Osteoclastogenic cytokines are represented by inflammatory cytokines such as tumour necrosis factor α (TNFα), interleukin-1 (IL-1), IL-6, and IL-8, and other interleukins such as IL-7, IL-11, IL-15, IL-17, IL-23, and IL-34. Anti-osteoclastogenic cytokines are instead represented by interferons (IFN), such as IFNα, IFNβ, and IFNγ and again interleukins such as IL-3, IL-4, IL-10, IL-12, IL-27, and IL-33. With the exception of TNFα, on activating the NFκB signalling pathway all other cytokines exert their biological effects through the cytokine receptor signalling pathway which uses, as intra-cellular transducers, members of the janus kinase (JAK) tyrosine kinase and the signal transducer and activator of transcription (STAT) transcription factor families.

Osteoclastogenesis is also influenced by a process known as macrophage polarization according to which macrophages can undergo an M1 (inflammatory) or M2 (reparative) functional activation. In this regard, it has been demonstrated that an increase of M1/M2 ratio favours osteoclastic differentiation [40,41].

Among chemical factors able to modulate osteoclastogenesis, the most important are represented by increased extra-cellular concentrations of H^+^ (acidosis) and Mg^2+^, both able to remarkably potentiate osteoclastic differentiation [16,42] and Zn^2+^, inhibiting the same process [43].

### 1.10. The Cross-Talk between Osteoblasts and Osteoclasts

Osteoblasts (OBs) are bone-forming cells residing in the bone tissue where they counteract the opposite activity of OCs. The interaction between osteoblasts and OCs is regulated by a complex network of stimuli. Besides promoting osteoclastogenesis, VD3 is also a powerful inducer of osteoblast differentiation. Upon exposure to VD3 stimulation, OBs also produce RANKL, favouring osteoclast differentiation [31]. At the same time, OBs produce an anti-RANKL soluble decoy receptor that is called osteoprotegerin (OPG) and inhibit RANKL activity. On the other site, OCs secrete a signalling molecule called Wnt able to enhance osteoblast differentiation acting through the β-catenin signalling pathway [32].

Acting together, OCs and OBs, participate to a process that is called bone remodelling cycle and is aimed to replace old/damaged bone with new/healthy one. This activity is divided in five steps: activation, resorption, reversal, formation, and termination. The first step is characterized by the activation of OCs, then leading to the resorption of bone that is accomplished during the second step. The third and most important step (also called the reversal phase of bone remodelling cycle) is needed by OCs to activate OBs, giving rise to the formation of bone occurring in the fourth step. This is a key moment of the process and, in addition to already mentioned stimuli secreted by OCs and acting on OBs (such as IL-6 and Wnt), it is thought to be mediated by ephrins, transforming growth factor-β(TGF-β), and bone morphogenetic protein-2 (BMP-2) [44].

### 1.11. Osteoclasts as Therapeutic Targets in Human Diseases

Several bone disorders such as osteoporosis and Paget’s disease (metabolic) or cancer bone metastasis and multiple myeloma (neoplastic) are believed to be favoured by a mechanism of osteoclast activation leading to a condition of increased bone turn-over. In these circumstances, inhibition of osteoclast differentiation can be to all effects considered as a powerful tool to cure the underlying disease. Drugs used to inhibit osteoclast activity are defined anti-resorptive agents (AR), due to their capacity to contrast bone resorption. Among AR drugs, the most important are represented by bisphosphonates (BPs) and denosumab (DMAb).

### 1.12. Bisphosphonates

This denomination is due to their chemical similarity with pyrophosphate with which the BPs share the presence of two phosphate groups inside the molecule. BPs can be divided in two classes: non nitrogen-containing and nitrogen-containing BPs [45].

Non nitrogen-containing BPs are represented by clodronate and etidronate. These compounds were discovered earlier but are less active and for this reason their use is substantially restricted to metabolic bone disorders. They exert an anti-resorbing effect that is obtained by competing with the recruitment of intra-cellular ATP in OCs, thus blocking the energetic reserve of these cells, inducing their apoptosis.

Nitrogen-containing BPs, such as zoledronate (ZA) and pamidronate, were developed later, exhibit a stronger activity, and are predominantly used in neoplastic bone diseases, but lower doses are also prescribed in benign conditions. The action mechanisms of these drugs have not been completely understood. However it has been demonstrated that they are up-taken by OCs where they inhibit the mevalonate metabolic pathway thus preventing Ras farnesylation, necessary to allow its anchorage to cell membrane and the consequent activation [46]. At the same time, they also inhibit c-src and PKC activity, therefore leading to a block of the M-CSF pathway (Figure 2). The final effect is similar to that observed with the non-nitrogen members of the family and is represented by the apoptosis of OCs. The persistence of nitrogen-containing BPs in bone has not been clearly quantified but it is supposed to remain there for a very long time due to a constant reuptake by new OCs [47].

### 1.13. Denosumab

Denosumab (DMAb) is a monoclonal antibody directed against RANKL and exerts its activity forming a complex with RANKL and thus inhibiting the activation of its downstream pathway [48]. Based on this premise, it is possible to consider that the action mechanism of DMAb mimics the activity of osteoprotegerin (OPG), a soluble anti-RANKL decoy receptor which is physiologically produced by OBs and inhibits OCs. A major difference, in comparison with BPs is therefore represented by the fact that DMAb is specific for the RANKL pathway (Figure 2). Furthermore, due to the different action mechanisms, DMAb is supposed to have a limited effect over time. The dose and the schedule of administration of DMAb varies depending on the bone condition, administered at a dose of 120 mg per month in cancer patients as compared with 60 mg twice a year in osteoporosis.

## 2. A Pathological Condition Related to Anti-Resorptive Agents: Osteonecrosis of the Jaw

As already explained in the previous section, OCs are particularly active in several skeletal disorders with high bone turn-over such as osteoporosis and Paget’s disease or bone metastatic cancer and multiple myeloma. For these conditions, AR, BPs, and DMAb represent a medical tool widely used in the supportive care for the prevention of bone fractures. The type of AR drug, the dose, the schedule and the potency are modulated on the type of bone disease.

Like any other treatment, even ARs are not exempt from adverse events, and since 2003 [49] BPs and DMAb [50] have been related to a condition called osteonecrosis of the jaw (ONJ), although a clear cause–effect relationship has not been completely demonstrated.

Interestingly, ONJ related to the use of BPs shares similarities with the historical phossy jaw induced by white phosphorus in the matchmaking industry.

Both BPs and DMAb target OCs, therefore, while the inhibition of osteoclastogenesis seems to be the most important mechanism involved, it might not be the only one.

Cases of ONJ have been related not solely to the use of AR, but also to anti-angiogenetic drugs and more rarely to other drug categories (i.e., tyrosine kinase inhibitors and m-TOR inhibitors; Figure 2) [51,52] supporting the concept that the pathogenesis might be multi-factorial.

Two key factors have been identified in anti-resorptive agents related osteonecrosis of the jaw (ARONJ): the potency of the drug and the duration of the treatment.

The importance of the potency has been extensively reported, and it can partly explain why ONJ is more frequently observed in the oncological setting compared with osteoporosis. The type of BP most frequently used in oncological and haematological patients is ZA, a highly potent nitrogen-containing BPs administered monthly. It is administered with a higher dose and frequency in these patients as compared to osteoporotic individuals. 

The long duration of the treatment has been also observed in retrospective and prospective studies, and after 2003 the escalating incidence of bisphosphonate osteonecrosis of the jaw (BRONJ) induced haematologists and oncologists to review the guidelines for the administration of BPs, limiting the therapy to an average of 2 years and reserving prolonged treatment to selected cases [53,54]. 

These two factors alone however do not explain the phenomenon, and other risk factors have been identified: invasive procedures, infections, impaired angiogenesis, and “patient” factors, as reported in the clinical section.

This review is focused on OCs and their involvement in the development of ONJ, so we will analyse the OC-dependent factors involved in the pathogenesis of ONJ, with a brief mention of other possible mechanisms.

### 2.1. Osteoclasts Dependent Factors

Gross et al. analysed jaw bone samples of patients affected by BRONJ, observing a peculiar histological pattern characterized by giant OCs (1.6 times larger than control) and hyper-nucleated (1.9 times more nuclei compared with control), a known characteristic induced by BPs that can be partly explained by the high expression level of DC-STAMP, a molecule involved in cell–cell fusion. However, the OCs were inactive from the resorptive point of view, as demonstrated by the low expression of TRAP+ OCs (a marker of OC bone resorption), the absence of the raffle border, and by the high number of OCs detached from the bone surface [55].

In a following study, Wehrhan et al. compared bone samples of patients affected by BRONJ with osteoradionecrosis, osteomyelitis, and normal samples [56] analysing NFATc1, an OC activator, and Bcl6, an OC suppressor. NFATc1 is part of the RANK–NFκB pathway, and is a key regulator of osteoclastogenesis and OC activity. Bcl6 is a suppressor of osteoclastogenesis and OC activity via NFATc1, DC-STAMP, and CSTK. The results, apparently paradoxical, showed that in BRONJ both NFATc1 and Bcl6 expression appeared to be increased. Wehrhan hypothesized that the increased expression of NFATc1 and therefore the activation of DC-STAMP could be a compensatory effect to the inhibition of the OCs secondary to the BPs.

The effect of AR agents on the development of ONJ might be explained as an interference on both OCs and OBs. It is well known that the bone homeostasis is regulated by both OCs, the key cells in bone turn-over, and OBs, in a process called coupling. A possible explanation of the role played by AR in the development of ONJ comes from a paper published in 2014 by Williams et al. [57] who developed a mouse model of ONJ in animals treated with ZA (BRONJ) and anti-RANKL MoAb (DRONJ). Both groups developed post-extraction ONJ-like lesions of the jaw that were related to the inhibition of bone remodelling, affecting the OC and OB coupling. Animals affected showed higher numbers of empty lacunae and necrotic bone, with serum concentration of TRAP-5b (the 5b serum isoenzyme of TRAP) markedly suppressed, in addition to suppressed alkaline phosphates (ALP) indicating the inhibition of both OCs and OBs. The inhibition of the bone remodelling was confirmed by the inhibition of new bone formation in the extracted socket in mice with ONJ-like. The pH of the bone environment might also contribute to the development of the ONJ, since it is recognized that acid milieu enhances catabolic activity-inhibiting OBs [58]. In an animal model Kim observed that rats treated with BPs and NH_4_Cl were more prone to developing ONJ after tooth extraction. The histomorphometric analysis showed that the bone architecture was more deteriorated compared with the alkalotic group, with a significant higher necrotic bone and clusters of empty lacunae, possibly related to the inhibition of the OBs activity in an acidic environment and increased catabolic activity [58].

However, the effect of the AR is not selective on the skeleton. Thus, how do we explain the typical localization at the jaw? Gong et al. [59] observed that ZA-treated rats showed an inhibition of bone remodelling in tooth extraction socket but enhanced bone repair in other skeletal sites. The effect of ZA was also tested in vitro in bone marrow stromal cells (BMSCs) collected from the tooth extraction socket and peripheral bone injury site: even in this case the cell proliferation and osteogenic differentiation was significantly inhibited in the cells collected in the jaw compared with other skeletal sites, possibly explaining the typical localization of ONJ. It has been observed in fact the accumulation of BPs is higher in the jaw compared with other skeletal sites due to the higher amount of hydroxyapatite and the high bone turnover [60,61]. These observations suggest that the cells in the craniofacial region are more sensitive to the treatment with ZA compared with other sites. In conclusions, BPs might favour ONJ formation affecting bone remodelling, through the inhibition of both OCs and also OBs in the coupling process.

### 2.2. Osteoclast-Independent Factors

#### 2.2.1. Invasive Procedures and Possibly Delayed Healing

Invasive dental procedures have been confirmed in several studies as another important risk factor [62]. Invasive procedures might increase the risk of infection of the tooth extraction socket. Several studies observed different types of bacteria, fungi, and viruses in the exposed bone of ONJ, but a recent study the microbiota of patients with or without ONJ did not find significant differences. More than the infection itself, it seems to be the altered immune response of the host that favours the development of the ONJ [63].

Among the immune cells a role in ONJ development seems to be played by altered neutrophils, γδT cells, and natural killer cells [63]. The immune cells and macrophages are involved in the wound healing process. In a mouse model it has been observed that ZA enhanced M1 macrophage polarization and reduction of M2 macrophages related to elevated toll-like receptor 4 (TLR4) expression, possibly interfering with wound healing [64]. In vitro studies demonstrated also that BPs alter macrophage viability and morphology [65].

#### 2.2.2. Angiogenesis

The anti-angiogenetic effect of BPs has been observed in several studies, mediated by an anti-proliferative effect of BPs on endothelial cells and impaired vascularization of the mandible in rats [66,67,68,69]. Allegra et al. demonstrated a reduced number of circulating endothelial cells progenitors increased apoptosis of endothelial cells in myeloma patients treated with BPs, leading to anti-angiogenesis and avascular necrosis [67]. The mechanism might be mediated by the inhibition of IL-17, a mediator of angiogenesis through vascular endothelial growth factor (VEGF) and TNFα [70]. Damage of the epithelial mucosa induced by BPs or sunitinib (a drug inhibiting several tyrosine kinase receptors) may have also a role in the development of ONJ with other co-factors [71].

## 3. Clinical Definition, Epidemiology, and Risk Factors for Osteonecrosis of the JAW

ONJ was described in 2003 [49] as painful bone exposure of the mandible, maxilla, or both, non-responsive to surgical and medical treatment in cancer patients receiving treatment with pamidronate or ZA. In 2010, DMAb-related osteonecrosis of the jaw (DRONJ) was observed [50]. In 2014 the anti-VEGF agent bevacizumab was also reported to induce ONJ [72]. In 2016 the anti-sclerostin antibody romosozumab in a phase III study was demonstrated to induce ONJ [73].

ONJ may stay asymptomatic for long periods, ranging from weeks to several months or years [74]. Typical clinical manifestations are painful and often infected areas of necrotic bone in maxillo-facial areas that can lead to severe chronic pain and facial soft tissue infections. This can impair eating and speaking of patients, and the quality of life of these patients rapidly decreases. 

The International Task Force on ONJ [74] defines ONJ as:

(1) Exposed bone in the maxillofacial region which does not heal within 8 weeks after identification by a health care provider;
(2) Exposure to an anti-resorptive agent (BPs or DMAb);
(3) No history of radiation therapy to the craniofacial region.

**Figure 3:**
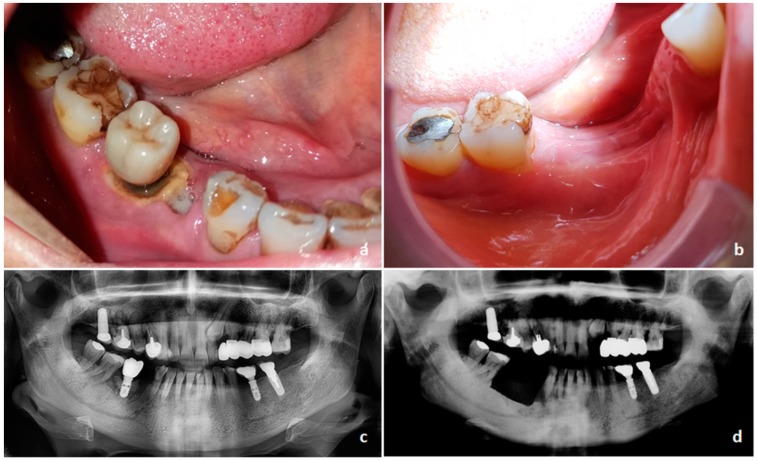
Clinical aspect of classical exposed bone variant of osteonecrosis of the jaw (ONJ). (**a**) Intraoral view of exposed and necrotic bone around a dental endosseous implant, together with infected post-extraction socket of dental element 45, with purulent discharge. Due to multiple myeloma, the patient had received zoledronate one year before. Extraction of tooth 45 was the trigger factor for ONJ. (**b**) Preoperative panoramic radiograph showing absent bone remodelling of the 45-extraction socket. (**c**) Postoperative healed intraoral view after a marginal mandibulectomy for removing the diseased bone. Primary closure of the surgical site was obtained by mobilization of a muco-periosteal flap. (**d**) Postoperative panoramic radiograph showing changes to trabecular pattern-dense bone of right hemi-mandibular body.

**Figure 4:**
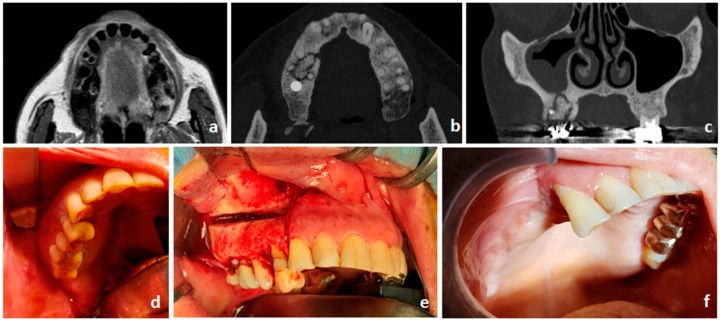
Clinical aspect of non-exposed bone variant of ONJ. The same patient from Figure 3 did not complete the recommended periodontal evaluation of dental elements of the upper right jaw. She developed a recurrent upper dental abscess, with swelling of the right cheek. This is a clinical manifestation of the non-exposed bone variant of ONJ. (**a**) Preoperative magnetic resonance imaging (MRI) showing high signal intensity of periodontal space in the right upper jaw. (**b**) Computed tomography (CT) scan in axial view showed large areas of increased medullary bone density of the upper dento-alveolar process. Bone sequestration was present close to periodontal disease. (**c**) CT scan in coronal view showed large areas of increased medullary bone density of the upper dento-alveolar process. Bone sequestration was present close to periodontal disease. Upper jaw involvement is related to a maxillary sinusitis. (**d**) Preoperative intraoral view with absence of exposed necrotic bone (unexposed variant of ONJ). (**e**) Intraoperative view of right partial upper maxillectomy. (**f**). Postoperative healed intraoral view. Primary closure of the surgical site was obtained by Bichat buccal fat pad transfer and mobilization of a muco-periosteal flap.

Evidence of exposed necrotic bone (E-ONJ) (Figure 3) is restrictive for ONJ definition according to the International Task Force, but the non-exposed variant (NE-ONJ) (Figure 4) has described by different groups of investigators and recently supported by a multicentre retrospective study [75]. Clinical features consistent with NE-ONJ include otherwise unexplained soreness in the jaws, fistula, loose teeth, swelling, and in advanced cases, pathological fracture of the mandible. Fedele et al. [75] described that use of alendronate, diagnosis of osteoporosis, and dental infection were more commonly found in patients with the NE-ONJ.

Despite the extensive ongoing research, the mechanism that induces ONJ is still unclear. The prevalence of BRONJ in patients taking oral BPs for the treatment of osteoporosis ranges from 0% to 0.04%. No information about the prevalence of DRONJ in patients receiving subcutaneous DMAb therapy for osteoporosis has been noted [74]. In ONJ epidemiology it has been documented that most cases (more than 90%) concern oncological patients receiving high doses of AR drugs [76]. The prevalence of BRONJ following tooth extraction in cancer patients receiving intravenous ZA is estimated to range from 1.6% to 14.8%; tooth extraction is the main triggering factor for the development of BRONJ in cancer patients [62].

A current systematic review [77] reported that the triggering factors of ONJ include:

(1) dental extraction (61.7%)
(2) spontaneous occurrence (14.8%)
(3) oral surgery (7.2%)
(4) prosthodontic trauma (7.4%)
(5) periodontitis (5.0%)
(6) dental implant treatment (3.9%)

Glucocorticoid therapy, diabetes, erythropoietin therapy, tobacco use, and renal dialysis have also been reported as concomitant medical issues.

Nevertheless, an increasing amount of data suggests that dental infection, instead of dental extraction in itself, might be the main local risk factor for medication-related ONJ [78,79]. This position was recently sustained by The European Task Force on medication-related ONJ [80]. The hypothesis supported by these data is that the osteonecrosis might arose within the alveolar bone due to intrabony periodontal infection, lacking mucosal fenestration and bone exposure [81]. Necrotic alveolar bone exposure could then develop or be a consequence of local triggering factors such as surgery or local trauma [75]. Because of dental/periodontal infection, a large part of ONJ patients had already developed NE-ONJ before the actual extraction occurred, and these cases should not be classified as E-ONJ [80]. Several scholars conclude that NE-ONJ and E-ONJ are manifestations of the same pathological entity [75,81,82,83].

As we have described above, the role of the active form of vitamin D3 (VD3) in the activation of OCs (and OBs) differentiation is described by several scholars [23,24]. A case–control study has recognized osteomalacia as a potential risk factor for BRONJ in oncological patients [84], which arises in the presence of VD3 deficiency [85]. Therefore, VD3 deficiency has been hypothesized by several authors as a risk factor for BRONJ in humans [86,87]. However, in a recent matched case–control study, patients with or without ONJ had the same frequency of VD3 deficiency and the same values of 25-OH-VD, parathormone (PTH), and most bone turnover markers. These results do not sustain the hypothesis that VD3 deficiency is involved in the pathogenesis of ONJ [87].

### Drug Holiday and Treatment

The half-life of BPs is longer than 10 years [47]. There is no evidence that the discontinuation of oral BPs is necessary before dental surgery [74]. Discontinuation of intravenous BPs and subcutaneous DMAb may be considered by oncologists [74]. However, no data are available for supporting the effectiveness of secession of intravenous BPs and subcutaneous DMAb on the prevention of ONJ. In a recent study, the discontinuation of DMAb was associated with reversal features of osteonecrosis in a mouse model [88]. Otto et al. suggested that any surgical intervention for ONJ needed to be suspended for at least several months after DMAb administration to avoid manifestation of ONJ [89]. Further study is needed to develop an appropriate strategy.

Oral rinses, pain control, limited debridement, irrigation, antibiotic therapy, and follow-up observations are the main recommendations by the American Association of Oral and Maxillofacial Surgeons [72]. However, a recent systematic review of therapeutic approaches found that ONJ rarely healed after these conservative treatments [90] On the contrary, clinical studies showed that surgeries to resect necrotic bone and/or surrounding healthy bone frequently healed ONJ [91]. Appropriateness of bone resection has to be determined preoperatively on the basis of computed tomography (CT) and magnetic resonance (MR), since the presence of osteomyelitis at one margin of resection was a predictor of ONJ recurrence [92]. Fluorescence-guided bone surgery has also been described [93]. Dental radiographs (i.e., panoramic radiography) are less informative with respect to other imaging methods in describing the extent of bony disease in ONJ [94]. According to published evidence, CT and MR appear to be superior over panoramic radiography in the investigation of ONJ [95,96], and we recommend these techniques in clinical treatment planning.

The osteotomies performed using piezo-surgical devices show more advanced stages of bone healing compared with rotary instruments [97]. After the bone resection, sharp bony edges were smoothened using burs, and tension-free wound closure was achieved using muco-periosteal flaps and the buccal fat pad if necessary; local flaps for closure with impaired vascularization have to be avoided [92,98,99]. When a pathologic fracture or ONJ involves the sinus, or when bony resection leads to a discontinuity defect of the mandible, microvascular composite tissue flaps have to be taken into consideration [74,100,101,102]. On the contrary, bone substitutes and grafting with bioactive bone substitutes have to be shunned [103,104,105,106,107,108,109,110].

As prevention strategy, most of available guidelines recommend oral examination and removal of unsalvageable teeth: all invasive oral procedures have to be completed with recovery before the start any anti-resorptive drug treatment. It has been demonstrated that the prevention reduces the risk of ONJ, although without eliminating it completely [74].

## 4. Conclusions and Hypothesis on Pathogenesis of Osteonecrosis of the JAW

The correlation between AR agents and ONJ has not yet been completely elucidated.

An attempt to explain the pathogenesis of ONJ, connecting the many actors possibly involved in the process, has been proposed by Di Nisio et al. [111] who studied the RANK/RANKL/OPG pathway in BP-treated patients. They hypothesized that the lipopolysaccharide produced by bacteria infecting the jawbone could possibly trigger the RANK/RANKL/OPG pathway, leading to OC activation in order to delimitate the infection. We could consider that the inhibition of OCs and OBs induced by the treatments, the altered host immune response, and possibly the impairment of angiogenesis and epithelial mucosa may result in an inability to limit the process, favouring the development of ONJ.

Based on these considerations, healing from ONJ probably requires a number of events, for example local re-activation of OCs (necessary to degrade necrotic/inflamed bone), recruitment of OBs (important to produce new healthy bone in place of the old and damaged bone), fibroblast proliferation and formation of new blood vessels (in order to support the entire process), and epithelial proliferation (to assure the rescue of mucosal integrity). Although all these steps are fundamental in the bone repair activity in ONJ, we suppose that the local re-activation of OCs could be the most important because it is the first part of the healing process and it could consequently allow the correct fulfilment of the others. It is plausible that a thorough comprehension of the biological aspects regulating bone remodelling cycle, with special regard to its reversal phase, might help to develop novel medical strategies to successfully cure ONJ as an alternative to surgical procedures.

## Figures and Tables

**Table 1:** Regulators of osteoclastogenesis. Table summarizing the main regulators of osteoclast differentiation classified as activators or inhibitors. IL: interleukin; TNF: tumour necrosis factor; IFN: interferon; OPG: osteoprotegerin.

Regulators	Activators	Inhibitors
Main Regulators	M-CSF, RANKL	OPG
Transcription Factors	NFATc-1; PU-1, C-FOS, MITF, TFE3	BCL-6
Modulating Signals	VD3, TNFα, IL-1, IL-6, IL-8, IL-7, IL-11, IL-15, IL-17, IL-23, IL-34	IFNα, IFNβ, IFNγ, IL-3, IL-4, IL-10, IL-12, IL-27, IL-33
Chemical factors	H^+^, Mg^2+^	Zn^2+^
